# Lysosomes and lysosome‐related organelles in immune responses

**DOI:** 10.1002/2211-5463.13388

**Published:** 2022-03-29

**Authors:** Colin Watts

**Affiliations:** ^1^ Division of Cell Signalling & Immunology School of Life Sciences University of Dundee UK

**Keywords:** antigen processing, endocytosis, immune response, lysosome‐related organelles, lysosomes, proteases

## Abstract

The catabolic, degradative capacity of the endo‐lysosome system is put to good use in mammalian immune responses as is their recently established status as signaling platforms. From the ‘creative destruction’ of antigenic and ‘self’ material for antigen presentation to T cells to the re‐purposing of lysosomes as toxic exocytosable lysosome‐related organelles (granules) in leukocytes such as CD8 T cells and eosinophils, endo‐lysosomes are key players in host defense. Signaled responses to some pathogen products initiate in endo‐lysosomes and these organelles are emerging as important in distinct ways in the unique immunobiology of dendritic cells. Potential self‐inflicted toxicity from lysosomal and granule proteases is countered by expression of serpin and cystatin family members.

## Graphical abstract

Lysosomes provide an environment that supports key events in the immune response including the processing of antigens for presentation on MHC molecules and the activation of some toll‐like receptors. Some leukocytes develop lysosome‐related organelles (LROs) to achieve their mostly cytotoxic effector functions. Mechanisms for protease regulation/neutralization by cystatin and serpin family members and immunodeficiencies impacting cytotoxicity are indicated by ‘stop’ signs.

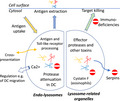

**Abbreviations**
AEP: asparagine endopeptidase
APC: antigen presenting cell
BCR: B‐cell (antigen) receptor
CF: cystatin F
CLIP: class II invariant chain peptide
CTL: cytotoxic T lymphocyte
DC: dendritic cell
ENU: N‐ethyl‐N‐nitrosourea
ER/ERAD: endoplasmic reticulum associated degradation
GILT: γ‐interferon inducible lysosomal thioreductase
Gmz: granzyme
HLA: human leucocyte antigen
IFNAR: interferon alpha receptor
Ii: invariant chain
LRO: lysosome‐related organelle
LSD: lysosome storage disease
MDA‐5: melanoma differentiation‐associated protein 5
MHC: major histocompatibility complex
MPS: mucopolysaccharidoses
MTOC: microtuble organizing centre
mTORC: mammalian target of rapamycin complex
NA: nucleic acid
NADPH: nicotinamide adenine dinucleotide phosphate
PAMP: pathogen‐associated molecular product
RIG‐I: retinoic acid‐inducible gene I
Serpin: serine protease inhibitor
SLC: solute carrier
TAP/TAPBPR: transporter of antigenic peptides (binding protein related)
TFEB: transcription factor EB
TLR: toll‐like receptor
TRPML: transient receptor potential cation channel, mucolipin family

The perception of lysosomes has undergone a remarkable change in recent years. Formerly viewed as end‐stage compartments dedicated to macromolecule catabolism enabled by a cohort of hydrolytic enzymes, which if missing or mutated could lead to lysosomal storage diseases (LSDs), they now occupy a prominent position in many other aspects of cellular and organismal physiology. In particular, lysosomes have emerged as key signalling platforms, as regulators of transcription, as agents of plasma membrane repair, in regulated cell death and in autophagy. These exciting developments have been extensively reviewed elsewhere [1, 2, 3]. Here we confine the discussion to the key roles that lysosomes and lysosome related organelles (LROs) play in immunity. Even with this restricted view their functions are broad. They play a key role in pathogen detection and signalling, in processing and presentation of antigens to T lymphocytes and are closely integrated into the different life stages of dendritic cells (DC) which are key cells that link innate and adaptive immunity [4, 5, 6]. Some leukocytes assemble specialised lysosome‐related organelles (LROs) which can be discharged at the cell surface to achieve distinct, mostly toxic, effector functions. Proteolytic enzymes, most with an acidic pH optimum, are important for many lysosomal functions in immunity. LROs accumulate distinct proteases and other toxic agents and we will see that potential toxicity from these organelles is countered by expression of members of the cystatin and serpin families of protease inhibitors. The term endo‐lysosomes rather than lysosomes is often used to refer broadly to later stage endocytic pathway organelles.

## Innate immunity: sensing and responding to pathogen products

### Endolysosomal processing of toll‐like receptors and their ligands

Innate immune responses occur rapidly after infection, providing some immediate protection and helping to start the slower acting adaptive response. These early responses include recognition by receptor systems, which collectively detect various pathogen‐derived molecular products (PAMPs)—distinct structural entities either not found in the host or not found in particular cell domains [7]. Toll‐like receptors (TLRs) are a key family, and while some TLRs operate at the cell surface, TLRs that detect viral and bacterial nucleic acids (NA), for example, double‐stranded RNA, are found within the lumen of endosomes and lysosomes. TLRs 3,7,8, and 9, which recognize different pathogen‐expressed nucleic acid configurations, fall into this group and are coupled through adaptors to kinase cascades that trigger cytokine production as well as developmental changes in the cell itself (reviewed in [8, 9]). By confining signalling to the endo‐lysosome system, activation should be limited to nucleic acids released following the breakdown of foreign pathogens since self‐DNA and RNA present in the external milieu will be destroyed by DNAse and RNAses. How is the activation of the nucleic‐acid sensing TLRs confined to the endo‐lysosomal pathway? It has been shown that endo‐lysosomal processing of both TLRs, and in some cases, their NA ligands is necessary as is the presence of the chaperone Unc93b1, which guides NA‐sensing TLRs from the ER to the endocytic pathway. A key early finding was that TLR9 underwent a lumenal proteolytic cleavage event and that only cleaved TLR9 engaged the cytosolic signalling adaptor MyD88 [10, 11].

Subsequent studies confirmed and extended the idea that proteolytic processing was required for the activation of TLRs 7,8 and 9. For example, TLR9 and TLR7 signalling in response to their specific agonists required processing by AEP in mouse dendritic and epithelial cells [11, 12]. The absence of AEP or mutation of the AEP cleavage site in the TLR7 lumenal domain blunted the production of inflammatory cytokines [12]. In other cells such as macrophages, cathepsins also contributed to TLR7 as well as TLR3 and 9 processing [13] and in retinal epithelial cells, TLR3 processing depended on cathepsins B and H and was required for signalling [14]. Other studies have demonstrated a role for the pro‐protein convertase furin in processing and activating TLR7 [15] and TLR8 [16] in some human cells. Unlike most endo‐lysosomal proteases, furin is active at neutral pH, and so, TLR7/8 cleavage might occur prior to arrival in the endocytic pathway. Thus, while the principal of TLR processing for foreign NA recognition seems universal, the precise proteases required vary somewhat between species and cell types. Importantly, the cleaved non‐membrane anchored TLR‐fragments remain non‐covalently associated and are still necessary for signalling (reviewed in [17]). Processing appears to be required not for ligand binding but rather for TLR oligomerization [18].

Large scale ENU mutagenesis screens in the mouse have identified additional players in NA‐driven TLR signalling [19]. As well as uncovering genes that are directly involved such as Unc93b1 [20], which chaperones TLRs 3, 7 and 9 from the ER to endosomes [20], this strategy and study of natural mouse mutants has also identified genes that are required for general endo/lysosomal integrity and trafficking, such as the AP3 adaptor and the amino acid and oligopeptide carrier SLC15a4, which is critical for TLR7 and 9 driven type‐I interferon (IFN‐I) production in plasmacytoid DCs and B cells [21, 22, 23]. Loss of SLC15a4 disturbed normal endo‐lysosomal v‐ATPase integrity and as a result pH regulation [24]. Kobayashi et al. provided evidence that this also impacted lysosomal mTORC1 signalling and disrupted the IFNAR‐mTOR‐IRF7 circuit that further boosts IFN production [24].

In the absence of DNAse II, undigested DNA activates cytosolic DNA sensors but not the endosomal sensor TLR9. It turns out that extended dsDNA such as bacterial DNA requires processing, by lysosomal DNAse II, to a 11‐12mer for TLR9 recognition [25]. The structures of TLR7/8 reveal binding sites for guanosine and oligoribonucleotides, indicating that lysosomal RNA degradation products rather than ssRNA itself activate this TLR. Indeed, two endo‐lysosomal RNases, RNase T2 and RNase A were required for TLR8 recognition of its RNA agonists [26]. Thus, lysosomal processing of both NA‐sensing TLRs and NAs themselves is required.

TLR4 can be activated by bacterial lipopolysaccharide but additionally by glycosaminoglycan fragments derived from extracellular matrix components. These accumulate in the mucopolysaccaridoses (MPS)—a family of lysosomal storage diseases (LSDs) characterised by inflammation and skeletal abnormalities. Simonaro et al. showed that TLR4 is activated under these conditions, in part due to lysosomal mislocalization, resulting in elevated serum TNFα levels. In a mouse model of MPS, the ablation of TLR4 or anti‐TNF therapy alleviated skeletal abnormalities [27]. These studies and others (reviewed in ref [28]) indicate that LSDs can lead to aberrant innate immune signalling.

### Endo‐lysosomes and cytosolic innate responses

While the TLRs operate at the cell surface and/or along the endocytic pathway, there are several sensors of PAMPs in the cytosol; for example, the NOD‐like receptors (NLRs), which recognize peptidoglycan motifs from bacterial cell walls, and the RNA helicases RIG‐I and MDA‐5, members of the so called RIG‐I‐like (RLR) family that recognise viral double stranded (dsRNA) and other distinct RNA species. Some of the ligands for NLRs and RLRs access these receptors through specific endo‐lysosome to cytosol transport mechanisms. In the case of the cytosolic NOD2 receptor, transport of the ligand muramyl dipeptide from endo‐lysosomes to the cytosol was shown to require the expression of solute carriers SLC15A3 and/or SLC15A4 in DC. NOD2 was recruited to SLC15A3/4 along with the kinase RIPK2 providing another instance of endo‐lysosomes as platforms for signalling [29]. Double‐stranded RNA is recognized by TLR3 in endo‐lysosomes but production of anti‐viral cytokines such as IFN‐I is also dependent on MDA‐5 and RIG‐I. Nguyen et al. [30] have shown that SIDT2 mediates transport of dsRNA from endo‐lysosomes to the cytosol. Importantly, this allows the anti‐viral response to be mounted and amplified by uninfected bystander cells and counters mechanisms developed by viruses to inhibit such responses in infected cells.

### Negative regulation of host defence by endo‐lysosomal proteases

As noted in the following section, the attenuation of endo‐lysosomal proteases often enhances rather than reduces MHC‐restricted antigen presentation since proteases create but can also destroy T cell epitopes. A similar negative effect of protease activity on host defence was shown by the finding that ablation of cathepsin B improved resistance to the intracellular pathogen *Francisella novicida*, which infects macrophages and other cells by escaping from phagosomes. In cells lacking cathepsin B, TFEB activity was elevated as was lysosome biogenesis and autophagy of *F*. *novicida*. Mechanistically, it was shown that cleavage of the Ca2+ channel TRPML1 by cathepsin B limited TFEB activity. The ablation of the protease increased the release of Ca2+ from lysosomes and activated TFEB via calcineurin mediated dephosphorylation of TFEB [31].

## Adaptive immunity: generating specific antibodies and T cells

### Antigen processing and presentation: class II MHC presentation

The adaptive immune response is slower to develop, but after several days, antigen specific B and T lymphocytes start to appear. B cells differentiate into plasma cells, which secrete large amounts of antibody and CD4 and CD8 T cells with distinct effector functions become established. Specialised subtypes of CD4 T cell are critical for activation and differentiation of other immune cells. For example, B cell development leading to high‐affinity antibody production and memory requires interactions with the so called T follicular helper cells (Tfh), while activation of macrophages harbouring bacterial pathogens is controlled by TH1 CD4 T cells and leads to microorganism killing. In both cases, presentation of foreign antigen in peptide form on the class II major histocompatibility complex (MHC) molecules of the B cell or macrophage is required to engage antigen specific Tfh or TH1 cells. These ‘effector’ T cells will have been previously activated following the presentation of the same antigen on a third type of antigen presenting cell (APC), the dendritic cell (DC). The activation of DC, particularly through the TLRs discussed above, sets in motion a developmental program that includes enhanced capacity to capture, process and present protein antigens. The molecular details of antigen processing and presentation on MHC molecules in DC and other cells have been intensively investigated and well‐reviewed [32, 33, 34]. Here, I provide a summary of some aspects relevant to the lysosomal focus of this volume.

### Outline of the ‘classical’ class II MHC pathway

The best studied route for antigens to be loaded on class II MHC involves their uptake into the endo/lysosomal pathway from extracellular sources by macropinocytosis, phagocytosis (including LC3‐associated phagocytosis) or receptor‐mediated endocytosis. At one or more locations, they encounter newly synthesised class II MHC, proteolytic enzymes and chaperones, mentioned below, to create a kind of ‘reaction vessel’ for peptide loading [35, 36]. Proteases generate antigen peptides and also prepare the class II MHC molecule for peptide binding. The class II MHC αβ dimer exits the ER chaperoned by the so called invariant chain (Ii), which acts as a surrogate peptide and targeting sub‐unit [37]. Removal of Ii can be initiated by different cathepsins and by AEP [38, 39], while the later stages of its stepwise removal require either cathepsin S or L [40], which leaves (a) a short fragment termed CLIP protecting the MHC binding groove and (b) the membrane anchor, which is degraded by the signal peptide peptidase SPPL2A [41]. CLIP is displaced in most cases by the action of a chaperone called DM (in humans HLA‐DM in mouse H2‐DM; reviewed in [42]). MHC molecules are highly polymorphic in mammalian populations with most of the allele to allele variation focussed on the peptide binding groove. At the population level, this broadens the range of pathogen‐derived peptides that can be presented to T cells but this feature also means that invariant CLIP binds with varying affinity and with varying dependence on DM for its removal [42]. DM, whose function in some cells is modified by a related protein DO, also acts as a peptide editor[43], removing low affinity peptides and ensuring that most peptides associate essentially irreversibly with class II MHC [44]. Peptide elution studies were first reported 30 years ago and have become increasingly sophisticated and comprehensive as mass spectroscopic techniques have advanced [45]. In a recent study, ~ 14 000 unique peptides were identified on class II MHC molecules expressed in a human DC cell line. The eluted peptides were mostly between 8 and 20 residues in length although peptides up to 30 residues were also identified [46]

One further player active in the endo‐lysosomal pathway should be mentioned. GILT (γ‐interferon inducible lysosomal thioreductase) is an oxidoreductase that reduces disulphide bonds in protein antigens. First shown to be required for optimal class II MHC presentation [47], GILT effectively aids antigen unfolding to augment protease action and has also been shown to operate in the class I MHC pathway of antigen cross‐presentation which is discussed below.

### Antigen processing and peptide capture by class II MHC

Processing of antigens that enter the class II MHC pathway relies on the same proteases responsible for Ii processing. Although requirements for specific enzymes can be found *in vitro*, to this author's knowledge, an absolute requirement for a specific endo‐lysosomal protease in class II MHC antigen presentation *in vivo* has yet to be observed. For example, AEP was necessary and sufficient for processing and presentation of a domain of the tetanus toxin antigen (TTCF) when human B and T cells were studied *in vitro* [48]. However, mice lacking AEP still mounted anti‐TTCF responses, albeit more slowly [49]. Lower levels of AEP in primary versus immortalized cells and the longer time scales of antigen presentation measured *in vivo*, explained this result [49]. When antigens are internalised on specific antigen receptors, for example, membrane immunoglobulin (mIg or BCR) on B cells, much lower levels of antigen suffice to activate a T cell response [50]. Alongside more efficient antigen uptake, more efficient transfer of processed antigen to receptive class II MHC may also occur because processing can begin before dissociation of antigen from the BCR and large antigen fragments are stabilized and partially protected from further processing and release into the endo‐lysosomal lumen is delayed [51]. Indeed, T cell epitopes in these fragments can be ‘handed over’ to neighbouring class II MHC molecules in a DM‐dependent reaction confined to the plane of the membrane [52]. A physical association between BCR and a sub‐set of class II MHC [53] and between BCR and DM [54] has been demonstrated, which could facilitate such a ‘handover’ mechanism.

Early capture of such tethered antigen fragments is likely to be advantageous compared to fragment release into the protease‐rich lumenal space not least because as noted above, T cell epitopes can be destroyed by protease action as well as generated as several studies have shown [55, 56, 57]. To offset this, early capture of processed antigen is desirable and is made possible by the fact that the peptide binding groove of class II MHC is open at both ends (unlike class I MHC), which allows the accommodation of long peptides or even partially unfolded proteins. There is now good evidence for such a ‘bind first trim later’ model of class II antigen capture first proposed by Sercarz and colleagues [58, 59].

Where along the endo‐lysosomal pathway does antigen processing and class II MHC/peptide complex formation take place? This was the focus of much early work by several labs. Immunoelectron microscopy of immortalized human B cells and later, dendritic cells, revealed striking multilamellar and multivesicular compartments rich in class II MHC molecules as well as DM, proteases, and other lysosomal markers [60, 61]. Significant cell type differences were observed and various naming systems applied but broadly, these compartments were located between peripheral recycling endosomes and terminal lysosomes. Use of subcellular fractionation, antigen and class II MHC radiolabelling and compartment ablation techniques all contributed to a picture of class II MHC/Ii delivery to early endosomes (even via the cell surface) and Ii processing and peptide loading in later endo‐lysosomal compartments [62, 63, 64, 65] reviewed in [33, 34] although antigen proteolysis can be initiated soon after uptake in specific B cells [51, 66]. Several studies have made the important point that different T cell epitopes can be loaded at different locations along the pathway with varying dependency on the DM protein [67, 68, 69]. Variations on the theme include the possibility of peptide exchange on recycling mature class II MHC molecules [70, 71, 72] and peptide loading at the cell surface following extracellular antigen processing using processing enzymes distinct from those found in endo‐lysosomes [45, 69].

Assembly of class II MHC/peptide complexes in compartments ‘late’ in the endocytic pathway with elaborate membrane morphology raised the question of how they are transferred to the cell surface. This could be achieved by direct fusion with, or by vesicle budding and transport to, the cell surface. Unless the internal vesicles fuse back to the limiting membrane prior to fusion with the cell surface [61], they will be released as so called exosomes, which can have amplifying or inhibitory effects on immune responses (reviewed in [73]). In DC, stimulation with TLR ligands induced tubulation of endo‐lysosomes containing peptide/MHC complexes [61, 74] as did DC contact with T cells specific for such complexes [75]. In both cases, these long tubules extended and made contact with the cell surface exemplifying another mode of delivery.

### Antigen delivery to lysosomes versus lysosome delivery to antigen?

Use of various soluble or particulate antigen preparations revealed the itinerary of antigen uptake, processing and class II MHC loading in endo‐lysosomes outlined above. However, an alternative scenario must be considered where B cells engage antigen immobilised on surfaces—a situation that is likely to be frequently encountered *in vivo* [76]. Remarkably, in this scenario, class II MHC positive endo‐lysosomes are directed and fuse at the contact site. This lysosome delivery to antigen rather than the other way round resembles the ‘immune synapse’ between cytotoxic CD8+ T cells (CTLs) and target cells discussed below and is driven by an analogous reorientation of the centrosome/MTOC requiring the GTPase Cdc42 [77]. Antigen may be extracted from a cell surface using mechanical force as a primary mechanism [78, 79]. However this can be aided by fusion of lysosomes at the site of antigen engagement. Release of lysosomal hydrolases and local acidification allows focalized antigen processing and extraction [77]. Class II MHC loading could then occur either at the cell surface in a ‘handover’ type reaction [52] or following endocytosis of extracted antigen and trafficking to internal class II MHC compartments.

How might lysosome fusion at BCR/antigen contact points be controlled? It has been shown in many cell types that wounded areas of the plasma membrane are rapidly repaired in a process dependent on extracellular Ca^2+^ and involving exocytosis of lysosomes. Repair seems to be achieved not by ‘patching’ but rather by the removal of damaged areas either via caveaolae and/or by membrane remodelling driven by the ESCRT complex (reviewed in [80]). Interestingly, B cell engagement with surface‐bound antigen seems also to trigger a lysosome‐mediated membrane resealing response. Maeda et al. [81] have recently shown that BCR signalling following contact with surface‐bound antigen induced transient membrane permeabilization and lysosome exocytosis whose extent was proportional to BCR/antigen affinity. It seems that an ancient membrane repair response has been co‐opted by the immune system to aid antigen processing and presentation by B cells with high affinity for surface bound antigens. Following ‘synapsing’ of the BCR with immobilized antigen, fusion of lysosomes required assembly of the exocyst, an evolutionarily conserved multisubunit complex involved in organelle/plasma membrane fusion events from yeast to mammals [82]. It would be interesting to investigate whether any of the hereditary conditions mentioned in the following section that impact on granule/LRO trafficking and fusion also affect targeting of class II MHC rich lysosomes to the B cell/antigen interface.

### Non‐classical class II MHC‐restricted antigen presentation

When the peptides captured by Class II MHC molecules are eluted from antigen presenting cells either *in vitro* or *in vivo*, mass spectrometric analysis demonstrates that the source proteins are not only from extracellular sources but also from intracellular compartments as well (reviewed in [45]). How do endogenous cytosolic and nuclear proteins get processed and loaded on endo‐lysosomal class II MHC molecules? Studies on different viral proteins have revealed different routes for endogenous antigens to access class II MHC molecules. First, autophagy has been shown in several studies to provide an access route to class II MHC, for example, following fusion of autophagosomes containing antigens with class II MHC‐positive compartments [83, 84]. An alternative route has been exemplified in studies on CD4+‐restricted responses to influenza antigens. A substantial proportion of the CD4+ T cell response is directed at antigens whose loading on class II is independent of autophagy but dependent on proteasomal processing. TAP in most cases was not required but in some instances optimal presentation did require GILT consistent with the disulphide bonded viral antigens studied [85]. These studies illustrate hybrid processing with contributions from the proteasome and endo/lysosomal hydrolases. How proteasome products of these biosynthesised viral antigens enter the endo‐lysosomal system in these studies is not yet clear. A recent study has shown that proteasomes, usually thought to be confined to the cytosol, can be found and are active within the lumen of endosomes and phagosomes [86]. Conceivably, this population may account for the proteasome‐dependence of endogenous antigens that are presented on class II MHC, but the issue of entry into endo‐lysosomes remains. Importantly, Eisenlohr and colleagues have shown that live viral vaccines (which can access this unconventional class II MHC pathway) elicited higher levels of protective antibodies compared with inactivated viral vaccines, which can only access the conventional exogenous class II MHC pathway [85].

### Antigen processing and presentation: cross‐presentation on class I MHC molecules

Classical antigen presentation on class I MHC molecules falls outside the scope of this review because peptide antigen is loaded in the ER rather than in endo‐lysosomes. Protein antigens that are manufactured in—or which can gain access to—the cytosol are generally the source proteins for class I MHC presentation. However, there are compelling reasons for allowing exogenous protein antigens, that is, those that access the endocytic pathway, to be presentable on class I MHC in some cell types. In particular, this is desirable in DC, which are the best initiators of class I MHC‐restricted CD8 T cell immune responses. This process called cross‐presentation ensures that class I MHC‐restricted immune responses can be made to pathogens, for example, viruses which do not infect DC.

### Features of the endo‐lysosomal pathway in DC which aid cross‐presentation

**Fig. 1:**
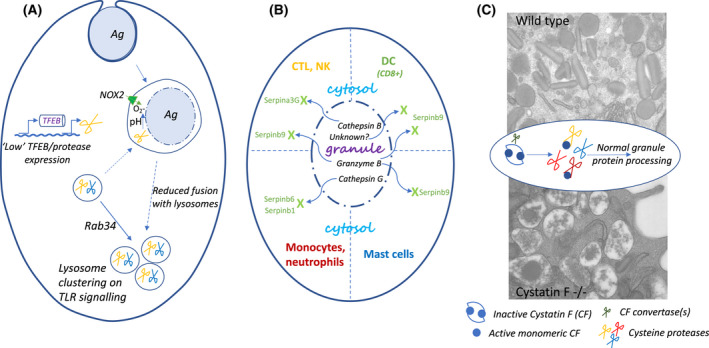
Control of and protection from protease activities optimises immune responses. (A) Dendritic cells (DC) utilize several mechanisms to attenuate protease activity to optimize antigen presentation on class I MHC molecules (cross‐presentation). Phagosomes containing antigen recruit NOX2 whose products raise lumenal pH and inhibit cysteine proteases. Fusion with lysosomes is suppressed through Rab34‐mediated, TLR‐signalling dependent lysosome clustering. Additionally, DC exhibit lower protease levels compared with macrophages due presumably to low TFEB activity. (B) Protease leakage from lysosomes/LROs is countered by cytosolic expression of different serpin family members. Granzyme B, in particular, is inhibited in different cell types by Serpinb9. In cross‐presenting DC, this serpin targets as yet unidentified enzymes. Other serpins inhibit misplaced cathepsins. (C) Cystatin F (CF) ensures normal granule biogenesis in eosinophils. CF is made as an inactive dimer, targeted to the endo‐lysosome/granule pathway and is activated by unidentified convertases to an active monomeric form, which can regulate several cysteine cathepsins. Electron micrographs show normal condensed granule content typical of eosinophils (top) and grossly disrupted and leaky granules in CF deficient cells (bottom). For more information and references, see the text.

The molecular cell biology of cross‐presentation has been extensively investigated and reviewed [87, 88, 89, 90] not least because protein‐based vaccines designed to elicit CD8 T cell responses (and DNA/RNA vaccines that do not express in DC) utilize this pathway. Early studies showed that exogenous antigens could be translocated to the cytosol to provide access to the canonical ER‐based and usually proteasome‐dependent class I MHC‐restricted pathway presentation pathway [91, 92]. How the translocation step is achieved has still not been fully established, but a variety of questions have been posed: is specific transport out of endosomes or phagosomes required or is simple rupture involved and if so, how is that triggered? Must antigens be partially unfolded (e.g., by GILT) and/or degraded for efficient translocation? Why are DC particularly good at cross‐presentation? Relevant to the last question is the fact, already mentioned, that endo‐lysosomal proteases can destroy as well as create T cell epitopes. Most of these enzymes have an acidic pH optimum. Early studies showed that cross‐presentation of pinocytosed antigens was enhanced when chloroquine, which alkalinizes acidic organelles and inhibits class II MHC presentation, was administered alongside antigen *in vitro* [91, 93]. Chloroquine increased the amount of antigen that appeared in the cytosol and remarkably, when individuals who had previously made responses to an HBV vaccine were boosted with the HBVEnv antigen, those additionally dosed with chloroquine showed strong recall CD8 T cell responses whereas those given antigen alone did not [93]. Later studies have shown that particular sub‐types of DC are particularly good cross‐presenters, at least in part because they are adept at preserving endocytosed or phagocytosed antigen (reviewed in [88]). Several distinct features of these DC have been discovered that allow antigen preservation (Fig. 1A). First, DC express lower levels of endo‐lysosomal proteases compared with, for example, macrophages [94]. Second, DC recruit the NADPH oxidase NOX2 to phagosomes leading to the introduction of reactive oxygen species, which attenuates proteolysis either by raising the lumenal pH [95] and/or by redox effects that specifically inhibit cysteine cathepsins [96]. The membrane components of NOX2 were derived from LROs, whose fusion with antigen containing phagosomes was dependent on Rab27a [97], analogous to Rab27a‐dependent CTL granule fusion discussed below. DC from Rab27a deficient mice showed greater phagosome acidification, enhanced antigen proteolysis and diminished cross‐presentation [97]. Third, DC activated by TLR signalling displayed a transient delay of fusion of phagosomes containing antigen with lysosomes to create a window of opportunity for efficient class I MHC peptide loading. Transiently delayed fusion correlated with perinuclear clustering of lysosomes, which was dependent on the GTPase Rab34 [98]. Fourth, non‐activated (immature) cross‐presenting DC had low levels of expression of the transcription factor TFEB, a key regulator of lysosomal gene expression and lysosome numbers [99]. Low TFEB and modest lysosome capacity again contributed to antigen survival and efficient cross‐presentation, whereas increased TFEB expression, induced by TLR signalling, increased protease expression and phagosome acidification consistent with earlier studies [100]. Under these conditions, cross‐presentation was reduced but presentation on class II MHC was enhanced [99]. This appeared to be due to enhanced levels of class II MHC expression on the cell surface and possibly to enhanced processing of the antigen although elevated protease activity does not necessarily favour presentation of antigens on class II MHC as already mentioned.

Further support for the finding that attenuated protease expression in DC can assist cross‐presentation came recently from an unexpected area. Mice lacking YTHDF1, a protein which binds to N6‐methylated adenosine residues (m^6^A) in mRNAs showed enhanced anti‐tumour CD8 T cell responses. Han et al. traced this result to improved cross‐presentation of tumour antigens on class I MHC molecules [101]. Mechanistically, YTHDF1 recognized m6A in several lysosomal cathepsin mRNA transcripts, boosting their translation, enhancing tumour antigen degradation and reducing cross‐presentation. The authors showed that antibodies targeting PD‐L1 (checkpoint inhibitors), to promote anti‐tumour immunity, were more effective in mice lacking YTHDF1 leading to the suggestion that this methylase could be a therapeutic target alongside checkpoint inhibitors [101]. Whether DC sub‐types differentially regulate YTHDF1 expression and/or the generation, reading and removal of this mRNA modification to achieve different antigen presentation outcomes is no doubt under investigation.

As noted above mutations in the Unc93b1 gene inhibit signalling through several NA‐sensing TLRs because of compromised TLR delivery to the endocytic pathway. The same mutation(s) also reduced class II MHC mediated presentation and even more strongly, cross‐presentation, independent of the effect on TLRs [20]. Antigen degradation and export to the cytosol were both reduced in Unc93b1 mutant cells [102]. Recent studies have revealed that Unc93b1 also partners the ER located Ca2+ channel STIM1 and have shown that STIM1 is also required for cross‐presentation [102]. STIM1 deficiency reduced antigen proteolysis and interfered with the phagosome/lysosome fusion events needed for the cross‐presentation pathway [102, 103].

### Antigen export from endo‐lysosomes and phagosomes to the cytosol

How do antigens exit the endocytic pathway in cross‐presenting DC? This has been a controversial area and much work done has been informed by the finding that the introduction of membrane from the ER into endo/phagosomes maybe a key facilitating step [104, 105, 106]. Since a lumen to cytosol retrotranslocation pathway operates in the ER as part of the ER‐associated protein degradation (ERAD) quality control system for newly synthesised proteins, it was envisaged that this machinery, if incorporated into antigen containing endo/phagosomes, might provide an exit route for antigens. Indeed, the suppression of ER incorporation into phagosomes, achieved by ablation of Sec22b, also inhibited cross‐presentation and in part, antigen export to the cytosol [107]. Unequivocal evidence that export to the cytosol requires a kind of ERAD‐type mechanism is however currently lacking. In an elegant study, Burgdorf and colleagues expressed a single‐chain fragment antibody (scFv) directed to Sec61—the translocon used for import into the ER of secretory and membrane proteins and which had also been proposed as the retro‐translocon element of the ERAD system [108]. This scFv also featured a KDEL ER retention signal, which selectively prevented Sec61 incorporation into endo/phagosomes but did not interfere with Sec61 function in the ER. Under these conditions antigen cross‐presentation was significantly suppressed. Sec61 therefore appeared to be a strong candidate for endo/phagosome to cytosol transport of at least some cross‐presented antigens [108]. However, more recent studies utilizing mycolactone as a fast‐acting Sec61 inhibitor challenge this view and indicate that it may not be the ERAD retro‐translocation channel nor the endosome to cytosol channel involved in cross‐presentation [109].

Other, translocon‐independent mechanisms for which there is some evidence invoke lipid peroxidation driven by NADPH‐oxidase (NOX2) generated reactive oxygen species, which lead to endo‐lysosomal membrane rupture [110]. Recent studies show that NOX2 can be activated following ligation of DNGR1 on the cDC1 sub‐set of murine DC [111]. DNGR1 recognizes actin as a marker of phagocytosable dead cell debris. Signalling via the tyrosine kinase Syk led to a NOX2‐dependent oxidative burst around DNGR1 positive phagosomes, membrane rupture and antigen delivery to the cytosol [111]. NOX2 mediated phagosome alkalinization on the one hand [95] and phagosome rupture on the other [111] would seem to be antagonistic processes, a point that perhaps requires clarification. Presumably, NOX2 mediated alkalinization would precede phagosome rupture and might conceivably aid rupture? For more details and discussion of yet more potential mechanisms for endo‐lysosome to cytosol antigen transfer, see [112].

### Alternative class I MHC loading scenarios in cross‐presentation

Translocation from endo/phagosomes to the cytosol, by whatever mechanism, achieves the objective of antigen delivery to the conventional ER‐localized class I MHC pathway. However, alternative scenarios have been demonstrated whereby proteasome processed or partially processed antigens are delivered back into ER‐phagosome hybrid organelles [104, 105, 106]. While some components come directly from the ER‐Golgi intermediate compartment (ERGIC) dependent on Sec22b function, others such as class I MHC itself can come from recycling endosomes dependent on Rab11a and stimulated by TLR signalling [113]. Trimming of peptide amino‐termini, which in the ER is performed by the ER aminopeptidase ERAP (ERAAP in the mouse), is performed in the endo/phagocytic pathway by insulin‐regulated aminopeptidase (IRAP). IRAP co‐localizes with class I MHC in endocytic compartments and its function is required for at least some cross‐presentation [114]. Where class I MHC is mature, that is, previously peptide loaded, peptide exchange may be important. TAPBPR, a relative of the PLC component tapasin and capable of exchanging peptides on some class I MHC alleles might be a candidate to support any peptide exchange needed in cross‐presenting compartments [115].

A distinct phagosome‐autonomous cross‐presentation pathway has also been demonstrated whereby the events of antigen processing and class I MHC loading take place solely within phagosomes. In this case, processing would be dependent not on the proteasome but rather on the protease systems discussed in the context of class II MHC antigen presentation. For example, cathepsin S was shown to be necessary for the presentation of class I MHC epitopes from several antigens [116]. A frequently cited caveat here is that the epitopes generated by lysosomal proteases are not likely to be the same as those generated by proteasomes and so the CD8 T cells elicited may not be useful for protection from viral infection. However, the phagosome‐autonomous pathway of cross‐presentation may have been given new life by the surprising finding of active proteasomes within phagosomes [86].

For more detail on the cell biology of DC cross‐presentation including important time‐resolved effects of TLR Signalling, see [87, 88, 89, 90, 117].

## Innate and adaptive immunity: lysosome‐related organelles in host defence

### Lysosome‐related organelles in cytotoxic leukocytes

Although a small fraction of lysosomes can fuse with the plasma membrane to aid repair following cell wounding [80], some cells generate more specialized ‘secretory’ lysosomes. These lysosome‐related organelles (LROs) include the granules of cytotoxic T cells, NK cells, mast cells, and eosinophils. Neutrophils assemble up to four distinct types of granules one of which, the azurophilic granule, contains lysosomal markers such as cathepsin G and CD63. However, these are considered specialized secretory granules rather than LROs. Immune cells that elaborate LROs package distinct effector molecules into the lumenal space and then direct these organelles towards the plasma membrane. In some cases, this involves a striking reorientation of the centrosome/microtubule organizing centre (MTOC) towards the plasma membrane, followed by ‘minus‐end’ directed movement of granules to the contact point with target cells [118]. A good example is the so called ‘immunological synapse’ made between a CD8 cytotoxic T cell (CTL) and a target cell displaying a specific class I MHC/peptide complex. Killing is achieved by delivery, via the pore‐forming protein perforin, of serine proteases called granzymes, which activate a caspase‐dependent pathway of cell death in the target cell. How are CTL granules, rich in perforin and granzymes (Gmz) targeted to the immunological synapse? The molecular cell biology of these events has been strikingly illuminated by the study of cells from patients with heritable immunodeficiencies. Examples include Hermansky‐Pudlak (HPS), Chediak‐Higashi (CHS) and Griscelli (GS) syndromes as well as some forms of familial hemophagocytic lymphohistiocytosis (FHL3‐5) [119, 120]. Although CHS appears to be caused by a defect in a single gene called Lyst, HPS and GS can be induced by defects at any of several genetic loci. The gene products are found in protein complexes that control intracellular trafficking including, in the case of HPS, the adaptor protein‐3 (AP3) and biogenesis of lysosome‐related organelle complex (BLOC1‐3) proteins. GS type 3 patients have a defect in the GTPase Rab27a and, among other conditions, a defect in the final steps of granule fusion in CTL and NK cells [119, 120]. Interestingly, AP3 defects in mouse models (*pearl* mice) and HPS2 patients also impact anti‐viral responses due to defective trafficking of TLRs 7&9 in plasmacytoid dendritic cells and consequently, reduced type 1 interferon production [21, 23, 121]. Further, loss of AP3 in *pearl* mice resulted in reduced TLR4 delivery to phagosomes and defective antigen presentation specifically from phagocytosed antigens [22]. FHL3‐5 patients also harbour mutations in proteins required for fusion of LROs/granules with the cell surface in CTLs, NK cells and platelets. Neutrophils, mast cells and eosinophils produce specialized granules, which are also likely to be affected by the mutations found in HPS,CHS, GS and other immunodeficiencies although limited data is currently available. For a comprehensive review of this area, see [119, 120].

### Serpin family members protect immune cells from toxic LRO content leakage

Some of the toxic effector proteins found in LROs and lysosomal proteases themselves are potentially dangerous to the host cell, for example, if leakage occurs into the host cytoplasm. To protect against this eventuality, cytotoxic leukocytes and some other cells utilize endogenous protease inhibitors from the serpin and cystatin families (Fig. 1B,C). Serpinb9 (Sb9, also known as Spi6) and Serpina3G (also known as Spi2A) are found in the cytosol of CTL and inhibit respectively the activities of GmzB and cathepsin B and potentially other targets. In the absence of these protective serpins, leakage of these proteases from CTL granules results in self‐inflicted toxicity and reduces either normal CTL expansion or establishment of CTL memory cells when Sb9 or Sa3G are missing respectively [122, 123]. Human serpinb9 is found in various human leukocytes and similarly protects CTLs from granzyme B [124]. Sb9 is also mainly responsible for protecting mast cells from the apoptosis inducing effects of GmzB released when granules are permeabilized with the lysosomotropic agent L‐leucyl L‐leucine methyl ester[125].

Sb9 is also expressed in some sub‐types of DC although its roles have been harder to pin down. DC engaged in triggering and expanding CTL might, similar to other cells, be targets for killing by GmzB, but reports differ as to whether Sb9 aids DC survival by inhibiting incoming GmzB [126, 127]. A distinct role of Sb9 in cross‐presentation by the CD8+ sub‐set of DC has been uncovered that is unrelated to DC survival. DC lacking Sb9 appeared to degrade internalized antigen somewhat faster, and as a result (see the section titled Adaptive immunity: generating specific antibodies and T cells above), were defective in cross‐presentation and CTL induction [128]. Interestingly, in contrast to the studies cited above in CTL [123, 125] and mast cells [125] where additional ablation of GmzB reverted the phenotype, this was not the case in this sub‐set of DC, indicating that Sb9 targets protease(s) other than GmzB (Fig. 1B).

Murine Serpinb1 and Serpinb6 are additional members expressed in the cytosol of monocytes and neutrophils where they inhibit granule localized proteases such as Cathepsin G (CatG). In mice lacking both these serpins, unrestrained CatG activity released from granules led to cell death by apoptosis and pyroptosis, the latter due in part to CatG activation of the pore forming protein gasdermin [129]. Doubly deficient mice also produced elevated levels of inflammatory cytokines such as TNF and IL1ß upon challenge with endotoxin [129].

### Cystatin F ensures safe LRO granule formation in eosinophils

The notion that leukocytes need to protect themselves from their own toxic granule contents is extended by evidence from mice lacking cystatin F (CF). The Type 2 cystatins are a family of inhibitors that inhibit members of the C1 and C13 cysteine protease families including the cysteine cathepsins (C1) and AEP (C13). CF is also known as leukocystatin due to its almost exclusive expression in leukocytes. Unlike related family members, which are mostly secreted, cystatin F is targeted to the endo‐lysosome pathway in an inactive dimeric configuration [130, 131]. Action by as yet unidentified convertase(s), generates active CF monomers, which are potent inhibitors of some of the cathepsins found in endo‐lysosomes [132]. This suggested that CF might act as a ‘buffer’ for protease activity in endo‐lysosomes and/or LROs: higher protease activity would activate more CF thus attenuating protease activity in a feedback fashion (Fig. 1C).

Eosinophil granules contain an array of proteins toxic to pathogens including large extracellular parasites such a helminth worms. However, the same proteins can also contribute to allergic disease, for example, in eosinophilic asthma. More recently, eosinophils have been shown to have tissue homeostatic and repair functions, mostly mediated through cytokine secretion [133]. Clearly, the toxic armoury found in eosinophil granules needs to be carefully packaged and regulated. We found that the integrity of eosinophil granules and indeed the viability of eosinophils was critically dependent on the expression of CF. Eosinophils in CF null mice had reduced lifespan, much reduced granularity and abnormal granule morphology [134]. This phenotype could be rescued with small molecule cysteine protease inhibitors indicating that regulation of one or more cysteine proteases (presumably cathepsins) is crucial for safe processing and packaging of toxic granule contents. In a model of allergic lung inflammation, CF null mice showed reduced lung pathology relative to wild type but conversely, clearance of the nematode parasite *Brugia malayii* was markedly compromised, both phenotypes due to reduced eosinophil numbers and granularity [134]. Targeting of CF to the endo‐lysosomal pathway is dependent on the mannose 6‐phospate/cation‐independent mannose 6‐phosphate receptor system, similar to most lysosomal hydrolases [135]. However, a proportion of dimeric CF is secreted and can be taken up *in trans* by neighbouring cells [135]. This raises the possibility that CF‐producing cells can regulate the endo‐lysosomal cysteine protease activities of non‐producing neighbours. Indeed uptake of CF into human CTL resulted in the suppression of multiple cathepsin activities, reduced granzyme activity and reduced killing ability [136]. Since some tumour cells express cystatin F [137], this might have been adopted as a strategy to evade a CTL response.

## Lysosomes, pinosomes and the integration of antigen processing and migration in DC

DC link innate and adaptive immunity by responding to pathogen products and by integrating into the response the key events of antigen capture, processing and presentation as well as migration from peripheral tissues to lymph nodes. The broader immunobiological features of DC that allow them to play this central role in immunity have been well‐reviewed elsewhere [4, 5]. Here, we summarize evidence that DC lysosomes play an important and unexpected central role in controlling these events. Earlier work had shown that antigen capture by DC showed a particular dependence on actin‐dependent macropinocytosis [138] and that sensing of pathogen products by TLRs (see above) could first stimulate [139] but subsequently suppress this pathway [138, 140, 141]. In vitro, macropinocytosis and motility in DC appeared to be mutually exclusive modes of operation [142] and *in vivo*, exploratory DC behaviour in the epidermis was similarly transiently arrested by pathogen sensing and capture [143]. These findings made biological sense allowing a focus first on antigen capture at the time and place of pathogen sensing and subsequently, a switch to antigen processing and DC migration. More sophisticated experimental systems developed by Lennon‐Dumenil and colleagues have uncovered new concepts and mechanistic detail in this area and notably, a central role for lysosomes. This group first linked the antagonism between endocytosis and motility to the distribution of myosin IIA. Enrichment of myosin IIA at the front of DC disrupted the front‐to‐back gradient of this motor protein required for migration and facilitated actin‐dependent macropinocytosis [144]. Remarkably, the class II MHC‐associated invariant chain was instrumental in maintaining the front‐of‐cell distribution of myosin IIA needed for macropinocytosis. Invariant chain destruction severed this link, suppressed endocytosis and re‐established the myosin IIA gradient required for migration [144, 145]. Lysosomes are important intracellular stores of Ca^2+^ and in DC TLR signalling triggered Ca^2+^ release through the channel TRPML1 [146]. Elevated cytosolic Ca2+ activates TFEB [147], which in turn sustains TRPML1 expression. Ca2+‐signalling driven by this positive feedback loop was shown by the same group to activate actin/myosin IIA activity at the rear of DC and to promote fast, directional motility [146]. Since macropinosomes have been shown to drive mTORC1 activity through bulk amino acid uptake [148], downregulation of this endocytic pathway in DC may initiate and/or amplify these effects through suppression of mTORC1 and consequently enhanced TFEB activation. Taken together, these studies show how lysosomes are integrated closely with other aspects of cell physiology and can orchestrate major developmental changes in cell behaviour critical, in this instance, for immunity.

## Conflict of interest

The authors declare no conflict of interest.

## Author contributions

CW wrote the article and prepared the Figure.
